# The burden of rheumatoid arthritis in the Middle East and North Africa region, 1990–2019

**DOI:** 10.1038/s41598-022-22310-0

**Published:** 2022-11-11

**Authors:** Seyed Ehsan Mousavi, Seyed Aria Nejadghaderi, Alireza Khabbazi, Mahasti Alizadeh, Mark J. M. Sullman, Jay S. Kaufman, Gary S. Collins, Saeid Safiri

**Affiliations:** 1 Student Research Committee, Tabriz University of Medical Sciences, Tabriz, Iran; 2 Department of Community Medicine, Faculty of Medicine, Tabriz University of Medical Sciences, Tabriz, Iran; 3 Research Center for Integrative Medicine in Aging, Aging Research Institute, Tabriz University of Medical Sciences, Tabriz, Iran; 4 School of Medicine, Shahid Beheshti University of Medical Sciences, Tehran, Iran; 5 Connective Tissue Diseases Research Center, Tabriz University of Medical Sciences, Tabriz, Iran; 6 Department of Internal Medicine, Faculty of Medicine, Tabriz University of Medical Sciences, Tabriz, Iran; 7 Social Determinants of Health Research Center, Tabriz University of Medical Sciences, Tabriz, Iran; 8 Department of Life and Health Sciences, University of Nicosia, Nicosia, Cyprus; 9 Department of Social Sciences, University of Nicosia, Nicosia, Cyprus; 10 Department of Epidemiology, Biostatistics and Occupational Health, Faculty of Medicine, McGill University, Montreal, QC Canada; 11 Centre for Statistics in Medicine, Botnar Research Centre, NDORMS, University of Oxford, Oxford, UK; 12 NIHR Oxford Biomedical Research Centre, Oxford University Hospitals NHS Foundation Trust, Oxford, UK

**Keywords:** Risk factors, Rheumatoid arthritis, Epidemiology

## Abstract

Rheumatoid arthritis (RA) is a chronic systemic autoimmune disease. The present study reported the burden of RA in the Middle East and North Africa (MENA) region from 1990 to 2019 by age, sex, and socio-demographic index (SDI). Publicly available data from the Global Burden of Disease (GBD) 2019 study was used to report the modelled point prevalence, annual incidence, and disability-adjusted life-years (DALYs) of RA, as counts and age-standardised rates with their corresponding 95% uncertainty intervals (UIs). In 2019, RA had an age-standardised point prevalence of 120.6 per 100,000 population (107.0–135.7) and an annual incidence rate of 5.9 (5.2–6.6) in MENA, which have increased 28.3% and 25.2%, respectively, since 1990. In 2019, the number of DALYs due to RA in the region was 103.6 thousand (74.2–136.7), with an age-standardised rate of 19.0 (13.9–24.9) DALYs per 100,000 population, which has increased by 18.6% since 1990 (6.7–28.2). The highest point prevalence was found in females aged 50–54, and in males aged 45–49. The highest number of DALYs was observed in the 50–54 age group. The MENA DALY rate was lower than the global rate (19.0 vs. 39.6 per 100,000), but the rate was higher in all age groups in 2019, when compared with 1990. In addition, from 1990 to 2019 an increased burden from RA was associated with an increase in SDI. In line with global trends, the burden of RA in the MENA region showed a steady increase from 1990 to 2019. This highlights the increasing need for updating the available health data to design more accurate guidelines to enable the early detection and treatment of RA in the MENA countries.

## Introduction

Rheumatoid arthritis (RA) is a chronic systemic autoimmune disease that is characterized by inflammatory changes in the synovial membranes. The main clinical manifestation of the disease is symmetrical polyarthritis of the small synovial joints of the hands and the feet, which spreads to larger joints as the disease progresses^1^. The exact etiology of RA remains unknown, although genetic, immunological, and environmental factors all appear to have a role in the pathophysiology of RA^2^. Multiple risk factors have been proposed for RA, including familial and genetic factors^3,4^, smoking and alcohol consumption^5,6^, insufficient physical activity^7^, high-fat diets^8^, lower socioeconomic status^9^, and occupational exposures^10^.

The Global Burden of Disease (GBD) study 2017 reported the global age-standardised prevalence of RA to be 246.6 (per 100,000 population) and the annual incidence rate to be 14.9 (per 100,000 population), demonstrating increases of 7.4% and 8.2%, respectively, since 1990. Globally, the point prevalence showed an increase with advancing age and these rates were highest in females aged 70–74 and among males aged 75–79 years old^11^.

The Middle East and North Africa (MENA) region encompasses a wide range of diverse ethnicities, cultures, lifestyles, and weather conditions, all of which may affect the burden of RA^12^. Therefore, the epidemiological pattern of RA needs to be explored for the individual countries that comprise the MENA region. In 2017, the age-standardised disability-adjusted life-years (DALYs) and annual incidence rates of RA were 36.7 and 15.0 per 100,000 population, respectively, in the MENA region^11^.

A study using World Health Organization (WHO) data reported the global burden of musculoskeletal diseases in 2000, 2010, and 2015, but did not specifically focus on RA and the estimates contained therein are now outdated^13^. Similarly, a study using GBD 2017 data reported the global, regional, and national burden of RA from 1990 to 2017, which also needs updating. Furthermore, most of the epidemiological patterns were presented at the global-level and so age and sex patterns of RA, and its attributable risk factors, have not been reported for several regions, including MENA^11^. Thus, there is a dearth of comprehensive studies specifically aimed at determining the burden of RA and its trends in the MENA region. Also, the rapidly changing economic, social and cultural landscape of the MENA region further underlines the importance of having a more detailed understanding of the disease. Therefore, the present study reported the most up-to-date data on the point prevalence, annual incidence, and DALYs of RA in the MENA region by age, sex and Socio-demographic Index (SDI), from 1990 to 2019.

## Methods

### Overview

The Institute of Health Metrics and Evaluation (IHME) conducts the GBD studies, which has collected data on the burden of 369 diseases and injuries from 1990 to 2019 in 204 countries and territories, seven super-regions and 21 regions^14^. The IHME is an independent global health research organisation that is based in the School of Medicine at the University of Washington. The IHME collaborates with partners across the world to produce timely, pertinent, and valid evidence about the global state of health. We used the publicly available GBD data, which were provided by the IHME and are available from the following website: https://vizhub.healthdata.org/gbd-results/. The countries located in the MENA region include: Afghanistan, Algeria, Bahrain, Egypt, Iran (Islamic Republic of), Iraq, Jordan, Kuwait, Lebanon, Libya, Morocco, Oman, Palestine, Qatar, Saudi Arabia, Sudan, Syrian Arab Republic, Tunisia, Turkey, United Arab Emirates and Yemen. A detailed description of the GBD 2019 methodology and the improvements made since 2017 are provided elsewhere^14,15^. Furthermore, more details on the fatal and non-fatal estimates can be found at https://vizhub.healthdata.org/gbd-compare/ and http://ghdx.healthdata.org/gbd-results-tool.

### Case definition and data sources

We provide a summary of the GBD methodology for RA. Whilst RA is a systemic autoimmune disease with articular and extra articular manifestations, the disability weights (DWs) used in GBD 2019 do not include extra articular disabilities. The 1987 American College of Rheumatology (ACR 1987) was used to define RA in GBD 2019^16^. ACR 1987 contains seven diagnostic criteria, which are described elsewhere^14,16^. Evidence collected from a systematic review was used in GBD 2019 to calculate the prevalence, incidence and mortality of RA. The exclusion criteria included: (1) studies with samples that were not nationally representative, (2) non-population-based studies, (3) those providing inadequate primary data on epidemiological parameters, (4) studies on a specific type of RA (e.g., seropositive RA) and (5) and review articles. Furthermore, studies using inpatient data were also excluded, since they would not be representative of the true prevalence^14^. GBD 2019 also included USA claims data for 2000, 2010–2012, and 2014–2016 (by state) and Taiwanese claims for 2016 that have not been included in the previous iterations of GBD^14^. RA mortality was estimated using data from vital registration, verbal autopsy and surveillance sources^17^. Data were excluded as outliers when they were implausibly high or low (relative to global or regional patterns), varied widely from established age or temporal patterns, or conflicted with other similar data sources from the same locations or locations^17^. The sources used to estimate the fatal and non-fatal burden of RA are available at: http://ghdx.healthdata.org/gbd-2019/data-input-sources.

### Disease model

Premature mortality from RA was estimated using the standard Cause of Death Ensemble model (CODEm), with the covariates included being shown in the appendix section of a recent GBD 2019 publication^17^. The prevalence, incidence and mortality data concerning RA were entered into DisMod-MR 2.1, which is a Bayesian meta-regression tool used for modeling and calculating estimates by pooling all available heterogeneous data in order to adjust for methodological differences and to examine internal consistency.

It was also assumed that there were no incidence or prevalence numbers in those younger than five years of age^14^. In order to improve the estimation of the range of case definitions, data from all sources were re-extracted by IHME. The ACR 1987 criteria^16^ was used as the reference for the other definitions, such as Rome 1961^18^, the American Rheumatology Association 1958^19^ or the European League against Rheumatism criteria^20^, which were identified using a single study covariate (non‐ACR_1987).

Further study covariates were added using data from administrative health systems, studies which covered regional instead of nationally/sub-nationally representative populations, and also for claims data.

### Severity and years lived with disability

The International Classification of Disease 10 (ICD-10) codes for RA (i.e. M05-M06 and M08) were used with three sequelae (severity levels), each of which had a specific DW, which ranged from 0.117 to 0.581 (Table S1). The DW values were calculated using the GBD 2013 European Disability Weights Measurement Study and the GBD 2010 Disability Weights Measurement Study^21,22^. The proportion of RA patients with each severity level was estimated using the Medical Expenditure Panel Survey (MEPS)^21^ and these proportions were used to divide the overall prevalence of RA into the three severity categories. Lastly, the severity-specific DWs were multiplied by the point prevalence of each severity category in order to produce years lived with disability (YLDs).

### Compilation of results

The years of life lost (YLLs) were estimated by multiplying the number of deaths in each age group by the remaining life expectancy in that age group, using information from the GBD standard life table. DALYs were calculated by adding together the YLDs and YLLs. Furthermore, 95% uncertainty intervals (UIs) were reported for each estimate, which were based upon the 25^th^ and 975^th^ values of 1000 ordered draws. All estimates were standardised using the GBD standard population, which is the non-weighted mean of the 2019 age-specific proportional distributions from the GBD 2019 population estimates for all national locations, except for populations lower than five million. The association between SDI and the burden of RA, in terms of DALYs, was produced using Smoothing Splines models^23^. SDI is a composite indicator that is based upon average income per capita, average years of schooling for the population older than 15 years of age and total fertility rate under the age of 25. SDI ranges from 0 (less developed) to 1 (most developed). The figures for the final estimates of the point prevalence and annual incidence rates were generated by R Software (V.3.5.2) using data available from http://ghdx.healthdata.org/gbd-results-tool.

### Ethics approval and consent to participate

The ethics committee of the Tabriz University of Medical Sciences approved the study (ID: IR.TBZMED.REC.1400.193). All methods were performed in accordance with the national guidelines and regulations.

## Results

### The Middle East and North Africa region

In 2019, there were 18.6 million (95% UI: 17.0–20.4) prevalent cases of RA globally, with an age-standardised point prevalence of 224.2 per 100,000 population (95% UI: 204.9–246.0), which represents an 8.1% increase since 1990 (95% UI: 7.5–8.6). Also in 2019, globally RA had an age-standardised incidence rate of 13.0 (95% UI: 11.8–14.3) per 100,000 and a DALY rate of 39.6 (95% UI: 30.5–49.5) per 100,000.

In 2019, there were 672.8 thousand (95% UI: 588.7–768.0) prevalent cases of RA in the MENA region, with an age-standardised point prevalence of 120.6 per 100,000 population (95% UI: 107.0–135.7), which represents a 28.3% increase since 1990 (95% UI: 25.5–30.9) (Tables 1 and S2). RA accounted for 36.5 thousand (95% UI: 32.1–41.6) incident cases in 2019, with an age-standardised rate of 5.9 (95% UI: 5.2–6.6), an increase of 25.2% since 1990 (95% UI: 22.4–27.7) (Tables 1 and S3). In 2019, the number of regional DALYs due to RA was 103.6 thousand (95% UI: 74.2–136.7), with an age-standardised rate of 19.0 (95% UI: 13.9–24.9) DALYs per 100,000 population, an increase of 18.6% since 1990 (95% UI: 6.7–28.2) (Tables 1 and S4).

**Table 1:** Prevalent cases, deaths and DALYs for rheumatoid arthritis for both sexes in 2019 and the percentage change in the age-standardised rates during the period 1990–2019. (Generated from data available from http://ghdx.healthdata.org/gbd-results-tool).

	Prevalence (95% UI)			Incidence (95% UI)			DALYs (95% UI)		
	Counts (2019)	ASRs (2019)	Pcs in ASRs 1990–2019	Counts (2019)	ASRs (2019)	Pcs in ASRs 1990–2019	Counts (2019)	ASRs (2019)	Pcs in ASRs 1990–2019
Global	18,583,481 (16955,383, 20433,859)	224.2 (204.9, 246)	8.1 (7.5, 8.6)	1,074,391 (975502, 1179332)	13 (11.8, 14.3)	6.5 (5.9, 7.1)	3,262,589 (2510208, 4091555)	39.6 (30.5, 49.5)	1.1 (− 4.3, 5.2)
North Africa and Middle East	672,827 (588698, 767,998)	120.6 (107, 135.7)	28.3 (25.5, 30.9)	36,548 (32078, 41,603)	5.9 (5.2, 6.6)	25.2 (22.4, 27.7)	103,572 (74242, 136655)	19 (13.9, 24.9)	18.6 (6.7, 28.2)
Afghanistan	19,298 (16168, 23019)	87.3 (75.9, 100.9)	14.4 (8.5, 20.9)	1338 (1138, 1586)	4.4 (3.8, 5.1)	12 (6.6, 18)	3205 (2238, 4279)	15.8 (11.4, 20.8)	19.7 (− 6.6, 42.1)
Algeria	43,094 (37004, 50172)	104.2 (90.2, 120.3)	30.3 (24, 37.3)	2294 (1983, 2670)	5.2 (4.6, 6)	31.3 (25.2, 38.2)	6391 (4426, 8496)	15.7 (11, 20.8)	26.4 (8, 46.3)
Bahrain	2606 (2230, 3105)	154.8 (135.9, 177.8)	36.1 (28.5, 43.6)	155 (132, 182)	8.1 (7.1, 9.2)	40.6 (32.4, 48.9)	421 (302, 563)	27.6 (20.4, 35.7)	48.3 (7.6, 83)
Egypt	84,338 (70531, 100823)	98.1 (83.8, 115.7)	29.8 (23.8, 36.6)	4763 (4071, 5605)	4.9 (4.2, 5.7)	31.1 (25.1, 37.2)	12,078 (7916, 16783)	14.2 (9.4, 19.5)	26.2 (8.9, 45.1)
Iran (Islamic Republic of)	99,141 (88570, 110731)	109.1 (97.8, 120.9)	9.3 (7, 11.8)	4684 (4128, 5303)	5 (4.5, 5.6)	7.6 (5.4, 9.7)	15,366 (10877, 20209)	17.3 (12.4, 22.7)	9.8 (− 3, 20.5)
Iraq	34,227 (28794, 41102)	101.2 (87.1, 119)	24.6 (18.2, 30.8)	2057 (1767, 2419)	5 (4.3, 5.8)	25.4 (19.7, 31.7)	5248 (3610, 7185)	16.1 (11.3, 21.8)	23.9 (3.5, 45.4)
Jordan	10,073 (8461, 12152)	102.1 (87, 120.5)	33.8 (27.4, 40.4)	586 (499, 693)	5 (4.3, 5.9)	34 (27.8, 40.4)	1487 (997, 2044)	15.5 (10.5, 20.8)	25.6 (9.5, 44.2)
Kuwait	5621 (4652, 6804)	113.3 (96.6, 133.9)	34.2 (27.4, 40.6)	307 (254, 373)	5.7 (4.9, 6.7)	32.8 (26.2, 39.1)	797 (524, 1109)	16.5 (11.2, 22.7)	40.8 (23.1, 60.9)
Lebanon	5965 (5034, 7036)	111.4 (94.3, 131.1)	40.3 (33.8, 48.1)	289 (246, 341)	5.4 (4.6, 6.3)	38.4 (31.8, 45.5)	894 (628, 1211)	16.7 (11.8, 22.5)	37.4 (14.8, 61.7)
Libya	6670 (5544, 8035)	94.1 (79.3, 111.6)	26.3 (20.9, 32.5)	356 (297, 426)	4.7 (3.9, 5.5)	25.5 (20.5, 31.3)	1012 (700, 1373)	14.7 (10.3, 19.6)	29 (6.7, 48.4)
Morocco	33,404 (28420, 39495)	90.7 (77.3, 106.8)	31 (25, 36.8)	1717 (1475, 2013)	4.5 (3.9, 5.3)	30.7 (25, 36.7)	5255 (3658, 7094)	14.6 (10.2, 19.5)	32 (9.4, 55.8)
Oman	3863 (3166, 4768)	94.6 (80.7, 111.8)	37.4 (31.1, 43.6)	239 (199, 295)	4.7 (4, 5.4)	37 (31.1, 42.7)	559 (376, 762)	14.3 (10, 18.7)	27.3 (6.9, 47.9)
Palestine	3561 (2991, 4281)	99 (85.1, 116.2)	19.4 (13.7, 25.9)	222 (190, 264)	5 (4.3, 5.7)	22.3 (16.3, 28.8)	566 (396, 755)	16.8 (11.9, 22.1)	20.4 (1.4, 42.9)
Qatar	3001 (2447, 3718)	103.2 (87.3, 121.4)	31.3 (24.7, 38.1)	185 (151, 233)	5.3 (4.5, 6.2)	33.2 (26.7, 40.3)	421 (280, 592)	15.1 (10.6, 20.6)	25.2 (9.6, 44.1)
Saudi Arabia	36,297 (29909, 44171)	98.9 (83.3, 117.1)	43.5 (37.5, 50.1)	2111 (1757, 2575)	5 (4.2, 5.8)	42.3 (36.9, 48.6)	5140 (3389, 7175)	14.2 (9.7, 19.5)	35.9 (14, 55.4)
Sudan	24,195 (20,326, 29,188)	83 (71.6, 97.1)	32 (26, 38.9)	1542 (1317, 1829)	4.2 (3.6, 4.8)	31.2 (25.2, 38)	3752 (2637, 5138)	13.4 (9.6, 17.9)	29.7 (7.8, 52.1)
Syrian Arab Republic	14,115 (11938, 16712)	98.7 (83.5, 116.2)	31.1 (25, 37.8)	741 (628, 871)	5 (4.2, 5.9)	32.9 (26.6, 40.2)	2073 (1416, 2802)	14.7 (10.1, 19.7)	26.4 (10, 45.8)
Tunisia	13,360 (11,354, 15,861)	101.2 (86.1, 120.3)	33 (26.5, 39.8)	633 (541, 737)	5 (4.3, 5.8)	32.8 (26.7, 39.1)	1993 (1378, 2743)	15.2 (10.4, 20.8)	32.7 (13.8, 54.5)
Turkey	203,556 (183966, 223605)	217.6 (197, 238.8)	41.8 (33.7, 49.6)	10,701 (9656, 11831)	11.4 (10.3, 12.5)	43.1 (34.9, 51.4)	32,813 (24231, 42624)	35.4 (26.2, 45.9)	16.2 (− 1.9, 34.6)
United Arab Emirates	10,061 (8235, 12561)	90.7 (76.8, 107.6)	24.3 (19, 30.6)	571 (455, 718)	4.8 (4.1, 5.6)	28.6 (23, 34.9)	1484 (973, 2034)	14 (9.6, 18.5)	21.5 (5.2, 39.3)
Yemen	15,698 (13140, 18944)	73.9 (63.5, 86.5)	18.8 (12.4, 25.9)	1019 (865, 1212)	3.7 (3.2, 4.4)	18.9 (12.9, 25.5)	2511 (1735, 3350)	12.5 (8.9, 16.4)	22.5 (− 2.8, 45.3)

*DALY* Disability-adjusted life-years, *UI* Uncertainty interval, *ASR* Age-standardised rate, *Pcs* Percentage changes.

### National level

**Figure 1:**
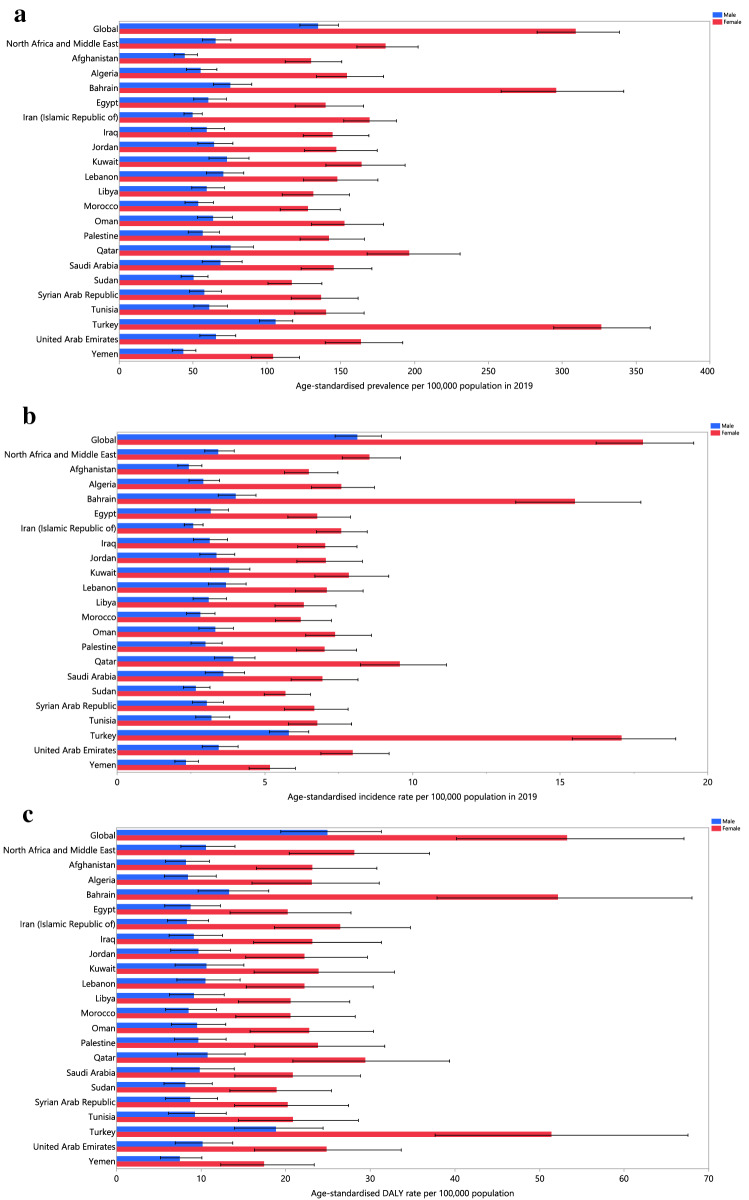
Age-standardised point prevalence (**A**), incidence (**B**), and DALYs (**C**) for rheumatoid arthritis (per 100,000 population) in the Middle East and North Africa region in 2019, by sex and country. *DALY* disability-adjusted-life-years. (Generated from data available from http://ghdx.healthdata.org/gbd-results-tool).

In 2019, the national age-standardised point prevalence of RA among the countries located in the MENA region ranged from 73.9 to 217.6 cases per 100,000 population. Turkey [217.6 (95% UI: 197.0–238.8)], Bahrain [154.8 (95% UI: 135.9–177.8)] and Kuwait [113.3 (95% UI: 96.6–133.9)] had the three highest estimates of age-standardised point prevalence of RA in 2019. In contrast, Yemen [73.9 (95% UI: 63.5–86.5)], Sudan [83.0 (95% UI: 71.6–97.1)] and Afghanistan [87.3 (95% UI: 75.9–100.9)] had the three lowest rates (Fig. 1A and Table S2).

In 2019, the national age-standardised annual incidence rates of RA among the countries that comprise the MENA region varied from 3.7 to 11.4 cases per 100,000 population. The highest rates were observed in Turkey [11.4 (95% UI: 10.3–12.5)], Bahrain [8.1 (95% UI: 7.1–9.2)] and Kuwait [5.7 (95% UI: 4.9–6.7)]. In contrast, the lowest rates were found in Yemen [3.7 (95% UI: 3.2–4.4)], Sudan [4.2 (95% UI: 3.6–4.8)] and Afghanistan [4.4 (95% UI: 3.8–5.1)] (Fig. 1B and Table S3).

In 2019, the national age-standardised DALY rate of RA among the countries located in the MENA region ranged from 12.5 to 35.4 cases per 100,000 population. The highest rates were observed in Turkey [35.4 (95% UI: 26.2–45.9)], Bahrain [27.6 (95% UI: 20.4–35.7)] and Iran [17.3 (95% UI: 12.4–22.7)]. Conversely, the lowest rates were seen in Yemen [12.5 (95% UI: 8.9–16.4)], Sudan [13.4 (95% UI: 9.6–17.9)] and the United Arab Emirates [14.0 (95% UI: 9.6–18.5)] (Fig. 1C and Table S4).

Increases in the age-standardised point prevalence, from 1990 to 2019, was observed in all the MENA countries, with Saudi Arabia [43.5% (95% UI: 37.5–50.1)], Turkey [41.8% (95% UI: 33.7–49.6)] and Lebanon [40.3% (95% UI: 33.8–48.1)] showing the largest increases over the measurement period (Table S2 and Fig. S1). There were no countries in the MENA region that showed a decrease in the age-standardised incidence or DALY rates of RA from 1990 to 2019. Turkey [43.1% (95% UI: 34.9–51.4)], Saudi Arabia [42.3% (36.9–48.6)] and Bahrain [40.6% (95% UI: 32.4–48.9)] showed the largest increases in the age-standardised incidence rates of RA over the measurement period (Table S3 and Fig. S2). In addition, Bahrain [48.3% (95% UI: 7.6–83.0)], Kuwait [40.8% (95% UI: 23.1–60.9)] and Lebanon [37.4% (95% UI: 14.8–61.7)] showed the largest increases in the age-standardised DALY rate over the same period (Table S4 and Figure S3).

### Age and sex patterns

**Figure 2:**
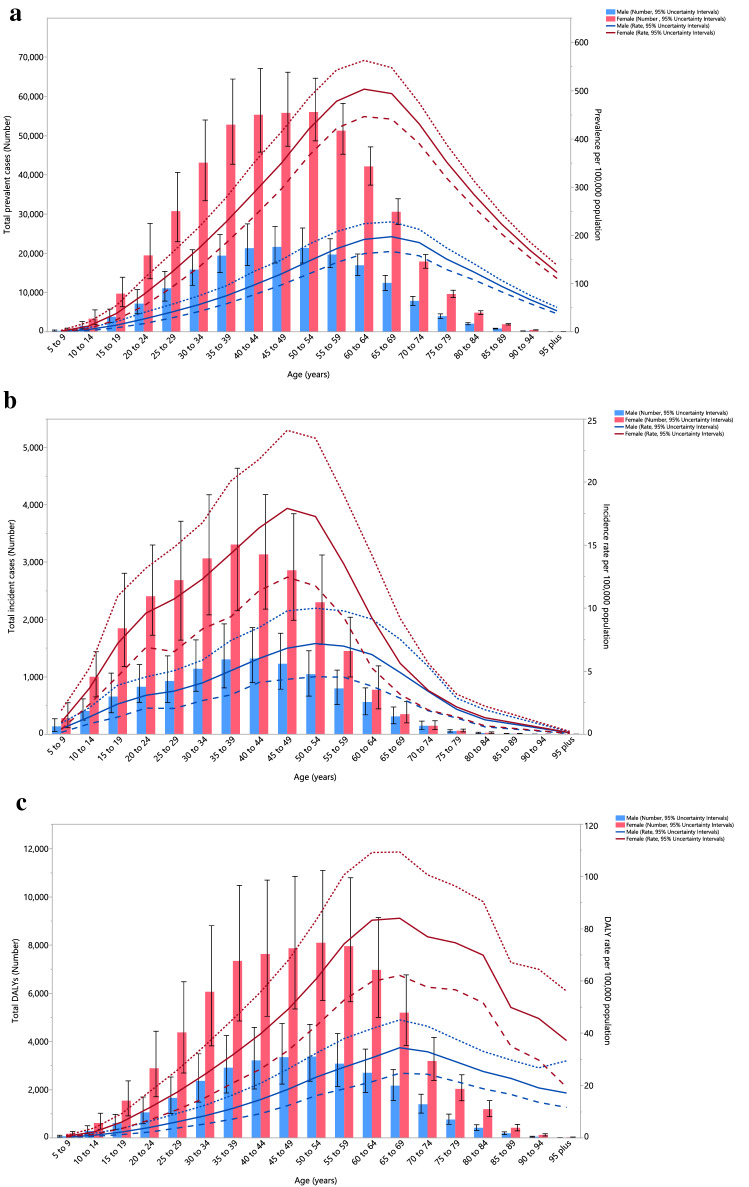
Number of prevalent cases and prevalence (**A**), number of incident cases and incidence rate (**B**), and the number of DALYs and DALY rate (**C**) for rheumatoid arthritis (per 100,000 population) in the Middle East and North Africa region, by age and sex in 2019; Dotted and dashed lines indicate 95% upper and lower uncertainty intervals, respectively. *DALY* disability-adjusted-life-years. (Generated from data available from http://ghdx.healthdata.org/gbd-results-tool).

In 2019, the regional point prevalence of RA was highest in the 60–64 age group in females and the 65–69 age group in males, but decreased from there to the oldest age group (95^+^). Similarly, the number of prevalent cases was highest in the 50–54 and 45–49 age groups in females and males, respectively, and then decreased with increasing age. Furthermore, the global point prevalence and number of prevalent cases of RA were higher in females of all ages (Fig. 2A).

In 2019, the regional incidence rate of RA was highest in females aged 45–49 and in males 50–54 years old. The number of incident cases peaked in the 40–44 and 35–39 age groups for males and females, respectively, after which there was a decline with increasing age (Fig. 2B).

There was a clear increase in the regional DALY rate of RA up to the 65–69 age group, followed by a decrease in the remaining age groups up to the oldest age group (95^+^). Females had a substantially higher DALY rate, with the highest number of DALYs being in the 50–54 years age group. The number of DALYs was higher in females, than males, in all age groups (Fig. 2C).

**Figure 3:**
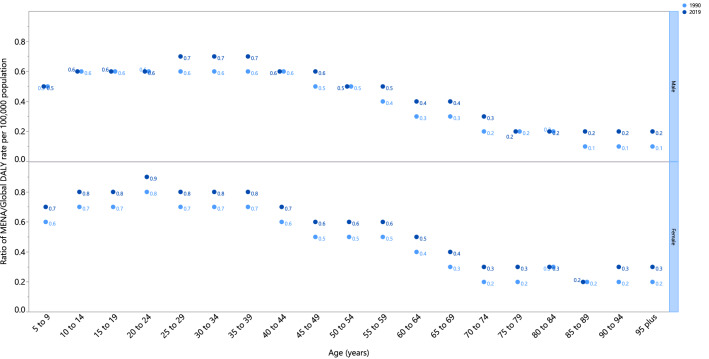
Ratio of the Middle East and North Africa region to global rheumatoid arthritis DALY rate according to age group and sex, 1990–2019. *DALY* disability-adjusted-life-years. *MENA* Middle East and North Africa. (Generated from data available from http://ghdx.healthdata.org/gbd-results-tool).

The rate ratio comparing the age-standardised DALY rates in MENA to global rates for different age groups, in 1990 and 2019, showed an increase up to the 25–39 age group in males and up to 20–24 age group in females, while the rate decreased thereafter in all age groups except for increases for females in the 90–94 and 95^+^ age groups. Although the MENA DALY rate was lower than the global rate, the DALY ratio for the MENA/Global rate of RA was higher in 2019 in all age groups than in 1990, meaning that the RA burden in the MENA region is heading towards the global level (Fig. 3).

### Association with the socio-demographic index (SDI)

**Figure 4:**
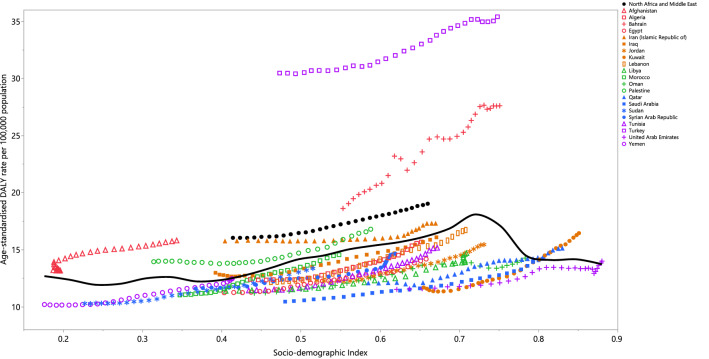
Age-standardised DALY rates of rheumatoid arthritis for 21 countries and territories in 2019, by SDI; Expected values based on the Socio-demographic Index and disease rates in all locations are shown as the black line. Each point shows the observed age-standardised DALY rate for each country in 2019. *DALY* disability-adjusted-life-years. *SDI* Socio-demographic Index (Generated from data available from http://ghdx.healthdata.org/gbd-results-tool).

In the period 1990 to 2019, the burden of RA generally increased with increasing socio-economic development, up to an SDI of around 0.7, but then decreased slightly as SDI increased. Countries such as Turkey, Bahrain, Iran, Palestine, and Afghanistan had much higher than expected burdens, whereas countries such as Sudan, the Syrian Arab Republic, Algeria, Lebanon, Egypt, Tunisia, and Libya had much lower than expected burdens (Fig. 4).

## Discussion

In the current study, the prevalence, incidence, and DALY counts and age-standardised rates were reported from 1990 to 2019 for RA in the 21 countries that comprise the MENA region, based on GBD 2019 data. Regionally, there were almost 672 thousand prevalent cases, 36 thousand incident cases and 103 thousand DALYs. Turkey, Bahrain and Kuwait had the highest national age-standardised prevalence and incidence rates, while the lowest national age-standardised prevalence and incidence rates were observed in Yemen, Sudan and Afghanistan. Turkey, Bahrain and Iran had the highest national age-standardised DALY rates, while Yemen, Sudan and the United Arab Emirates had the lowest national age-standardised DALY rates.

Previous studies have generally focused on describing global trends of RA and reporting the worldwide burden of the disease^11,24^, with the occasional small scale national report^25–32^. Therefore, there is a lack of comprehensive studies aimed specifically at analyzing the epidemiological patterns and burden of RA in MENA. In this study, we took an in depth look at the epidemiology and burden of RA in the countries that comprise the MENA region.

GBD 2019 shows that in the MENA region, RA was responsible for 103 thousand DALYs, which is different from the 187 thousand regional DALYs reported by GBD 2017^11^. In addition, the number of prevalent and incident cases in this region, 673 thousand and 36 thousand, respectively, were much lower in our study than that found in GBD 2017, which reported 1.3 million prevalent cases and 83.7 thousand incident cases in MENA^11^. The age-standardised point prevalence, annual incidence and DALY rates observed in GBD 2019 were 120.6, 5.9 and 19.0 per 100,000 population, respectively, which were also much lower than those estimated in GBD 2017, which were 259.3, 15.0 and 36.7 (per 100,000 population, respectively) in MENA^11^.

As for the changes in the regional trends between 1990 and 2019, the age-standardised point prevalence, annual incidence and DALY rates have increased by 28.3%, 25.2% and 18.6%, respectively. These findings were in line with the GBD 2017 report of 13.3%, 12.8% and 6.0%, respectively, which did not show any substantial differences^11^. However, any disparities observed in the findings of the different GBD studies can mostly be attributed to the inclusion of additional data sources and the use of new methodologies in more recent GBD iterations. Furthermore, the elevated incidence and prevalence of RA may also be related to an increased awareness of RA^12^.

The present study also revealed that for both males and females, the point prevalence rates and the number of prevalent cases, as well as the annual incidence rates and the number of incident cases, increased steadily with increasing age, peaking between the 6^th^-7^th^ and the 4^th^-5^th^ decades, respectively. After the peak, the numbers declined steadily across the older age groups. Furthermore, the number of DALYs was higher in females (than males) across all age groups. The age and sex patterns observed in our study were consistent with the findings of GBD 2017, which reported that globally females and individuals aged 70–74, had the highest burden of RA^11^. These findings highlight the importance of targeting females and the elderly with primary and secondary preventive programs.

In the present study, we also examined the relationship between the development level and the burden of RA in the MENA region. GBD 2019 shows an overall positive association between the burden of RA and SDI, up to an SDI of 0.7, after which the association was reversed. Previously, GBD 2017 reported a linear association between the burden of RA and SDI in the MENA region^11^. It is important to note that the association between the burden of RA and SDI was not simple to interpret, since the constituent countries of MENA had lower or higher than expected burdens.

At the national level, a 2018 study conducted in Turkey, which used the 1987 ACR criteria for RA, reported the age-standardised point prevalence of RA for the general population to be 0.56%, which is higher than our estimates based on GBD 2019 findings (0.21% (95% CI: 0.21–0.25)^25^. In a 2012 study carried out in Iran, which used the COPCORD Core Questionnaire (CCQ) to screen 19,786 people, the crude prevalence of RA was observed to be 0.37% (95% CI: 0.29–0.46), which is again higher than our estimates based on GBD 2019 data (0.12% (95% CI: 0.11–0.14)^27^. In addition, a 2010 study conducted in Algeria, on 125,253 individuals using the 1987 ACR criteria, reported that a prevalence of RA of 0.13% (95% CI: 0.10–0.17) in Barika, Algeria^31^. This study also calculated the prevalence of RA to be 0.15% for the total population of Algeria^31^, which is not substantially different to our findings (0.11% [95% CI: 0.09- 0.13]). The reasons for the disparities between our findings and those from previous national studies are likely to be due to sample size restrictions and methodological differences.

The overall findings from the present study, as well as the findings from previous GBD reports, highlight the ever-increasing burden of RA in the MENA region. As a result, the need for early detection and treatment strategies, along with effective preventive measures, is even more important than ever. One approach for controlling RA is to focus on modifiable risk factors. Previous research has reported a number of risk factors for RA, with cigarette smoking being the most important modifiable risk factor by far, since smoking has a clear association with RA^5^. Smoking cessation has been shown to reduce the risk of RA over time, although it does not have an immediate effect. In other words, twenty to thirty years after quitting, ex-smokers still have modestly higher rates of RA when compared with lifetime nonsmokers^33^. Therefore, policies to control and decrease smoking are of particular interest in the prevention of RA. Globally, between 1990 and 2019, the age-standardised point prevalence of smoking decreased by 27.5% in males and 37.7% in females^34^. In the MENA region, during that same period, a decrease in smoking was most evident among the male population (11.2%), but was also seen to a lesser extent in females (2.9%)^34^. In 2019, Turkey, which had the highest point prevalence and annual incidence rates in our study, was among the top ten countries for the proportion of tobacco smokers^34^. Afghanistan, which is among the countries with the lowest point prevalence and annual incidence rates of RA in the region, had a low age-standardized point prevalence of smoking in 2019, although the rate has increased rapidly by 179% and 205% in females and males, respectively, since 1990, which is also in accordance with the increases in the point prevalence and annual incidence of RA in this country^34^. Conversely, Bahrain has the second highest national age-standardized point prevalence and annual incidence rates for RA in the region, but the age-standardised smoking prevalence were not among the highest rates in the region and smoking has declined substantially since 1990^34^. Furthermore, research has shown the smoking attributable burden of RA to be 9.6%, with the remaining burden being comprised of other unknown factors^15^.

Depression has been found to be the most prevalent comorbidity of RA^35–37^. For example, a UK study of 4187 RA patients found that after five years 23.7% of men and 35.5% of women had been diagnosed with depression^38^. The predictive factors for depression among RA patients include high tension, low self-esteem, passive coping, fatigue, pain^39^, and female sex^38^. In addition, higher levels of rheumatoid factor, low income and low levels of mental health have been shown to be associated with depression in patients with RA^40^. Furthermore, serum interleukin-17 levels have been found to be significantly higher in RA patients with anxiety, compared with healthy controls and RA patients without anxiety^41^. Moreover, clinical depression raises the risk of mortality in RA patients, with a hazard ratio of 2.2^42^, which highlights the substantial effect of depression on the burden of RA. Interestingly, it seems that the association between depression and RA is bidirectional^43–45^. Previous studies have found a higher incidence rate of RA among depressed patients (compared to non-depressed controls) and a higher rate of depression among RA patients, compared to those without RA^46,47^. Furthermore, it appears that antidepressants may protect against developing RA^47^. In conclusion, screening programs for the early diagnosis and treatment of depression among RA patients should be developed to control and reduce the burden of RA.

Another important, albeit non-modifiable risk factor for RA, is old age. The MENA region is one of the seven super-regions that have seen increased life-expectancy at birth over the past seven decades^17^. In addition, the countries in the MENA region are considered to be in the mid-transition stage, where the crude rate of birth is only just beginning to fall, meaning that MENA countries have large and still growing populations, and as their populations age they will have an increased burden of RA^17^.

Moreover, the burden of RA in the region is also affected by disease severity. For that reason, strategies aimed at reducing disease severity are another potential approach for lowering the burden of RA in the MENA region. Disease severity in RA is assessed objectively using a disease activity score, which is based on 28 joint assessments (i.e., Disease Activity Score—DAS28)^48^. Studies show that lower Gross Domestic Product (GDPs) per capita is associated with higher DAS28 scores in RA sufferers^49,50^. Also, in countries with lower GDPs, the level of access and use of biological disease-modifying antirheumatic drugs (DMARDs) is much lower than in high GDP countries (7.5% vs 25.0%)^50^. Biological DMARDs are believed to be more effective for controlling RA, compared with conventional synthetic DMARDs^51–53^. Over the years, many studies have suggested that the observed association between low socioeconomic welfare and high DAS28 (in poorer countries) was due to the low use of biological DMARDs^54–56^. However, a more in-depth study has demonstrated that the intake of biological DMARDs is only responsible for 6.7% of the association between low GDP and high DAS28 scores^49^. These findings clearly indicate that not all of the improvements in DAS28 in high GDP countries can be explained by their access to better medication, instead other factors related to healthcare systems might be responsible for the observed patterns, including higher coverage of specialised rheumatological care, early referral strategies and tight disease control^49^.

Another obstacle specific to the region and individual countries, is the poor implementation of standardised rheumatological care guidelines^57^. For example, the new American College of Rheumatology and European League Against Rheumatism (ACR/EULAR) criteria, is accepted by many regional rheumatological associations and has the added benefit of including early RA diagnostic criteria, which can lead to an improved prognosis by initiating early diagnosis and treatment^24^. However, despite the relevance and applicability of the EULAR guidelines, the use of these guidelines in clinical practice in MENA is low, due to the perceived cost of these strategies^57^. Therefore, it has been suggested that local guidelines be designed which are specific to the needs, resources and challenges in each county, which can help to ensure an evidence-based approach in rheumatological care and to improve patient outcomes by facilitating more timely access to medication and specialised care^57^. Several recommendations have been published in order to address this issue^26,58^. Additionally, it has been suggested that data needs to be collected about the prevalence and epidemiology of RA in these countries, and that healthcare professionals, insurance companies and service providers, as well as patients, need to be educated about the burden of RA and the indirect costs arising from the suboptimal management of the disease^57^.

The cultural background of many countries in the MENA region means that patients are more inclined to seek help from herbal remedies and alternative medicines, rather than referring to specialist medical care^59,60^. This means that even in some of the more wealthy MENA countries, where medication and specialist care is accessible for free or at very reasonable prices, the timely management of the disease is still hindered by patient preferences and health literacy^59^. Finally, the necessity of RA prevention and control strategies should not be judged solely based upon the observed rates, but also on the burden of the disease that are anticipated in the future in the MENA region.

### Strengths and limitations of the study

To our knowledge, no previous study has comprehensively reported the burden of RA and the trends by demographic variables in the MENA region. Consequently, one strength of this study is that it used the most up-to-date evidence to estimate the burden of RA in the MENA region. However, our study also has the same limitations reported in previous GBD publications^15,17^. It should also be noted that due to the limited availability of population-based national data on the incidence and prevalence rates, in some countries the estimates presented here were based on models produced in DisMod-MR 2.1. Model performance may vary from country to country, depending on data availability, but the goal is to make as accurate an estimate as possible, by employing data from countries that are in a similar situation. Therefore, the national estimates presented here should be viewed with some degree of caution, especially those for countries like Afghanistan, Sudan and Yemen, which have been dealing with the devastating effects of war and social unrest. Also, owing to the effects of biological DMARDs in ameliorating the severity of RA and improving disease prognosis^51^, the availability of these treatment options should be taken into consideration in estimating the DWs and severity distribution within the different countries and regions^24^. Using the 1987 ACR criteria, instead of the new ACR/EULAR criteria which includes early symptoms of RA, may lead to an underestimation of the actual burden of RA, especially in regards to milder forms of RA. Therefore, the new ACR/EULAR criteria appears to be a better tool for estimating the actual burden of RA in future studies^24,61^. Also, GBD 2019 did not include the extra-articular symptoms of RA, which can affect the DW. Therefore, we suggest the modification of the DWs in further GBD iterations, based on extra-articular symptoms^24^.

## Conclusions

The burden of RA in the MENA region shows a steady increase from 1990 to 2019, and is expected to reach the global average levels in the upcoming years. This highlights the increasingly urgent need to update the available health data in order to design more accurate guidelines for the early detection and treatment of RA in the countries that comprise the MENA region.

## Supplementary Information

Supplementary Legends.

Supplementary Figure S1.

Supplementary Figure S2.

Supplementary Figure S3.

Supplementary Table S1.

Supplementary Table S2.

Supplementary Table S3.

Supplementary Table S4.

## Data Availability

The data used for these analyses are all publicly available at http://ghdx.healthdata.org/gbd-results-tool.

## Abbreviations

RA: Rheumatoid arthritis
DALYs: Disability-adjusted life-years
GBD: Global burden of disease
SDI: Socio-demographic index
MENA: Middle East and North Africa
UIs: Uncertainty intervals
ACR: American College of Rheumatology
EULAR: European League Against Rheumatism
CCQ: COPCORD core questionnaire
YLLs: Years of life lost
YLDs: Years lived with disability
MEPS: Medical expenditure panel survey
CODEm: Cause of death ensemble model
DWs: Disability weights
IHME: Institute for Health Metrics and Evaluation
WHO: World Health Organization
DMARDs: Disease-modifying antirheumatic drugs
DAS28: Disease activity score, based on 28 joint assessments
