# Supplementary material for: The burden of rheumatoid arthritis in the Middle East and North Africa region, 1990–2019
Source: Sci Rep. 2022 Nov 11;12:19297. doi: 10.1038/s41598-022-22310-0 (PMC9652423; doi:10.1038/s41598-022-22310-0)
Supplement: Supplementary file 1 — Supplementary Legends. [file 41598_2022_22310_MOESM1_ESM.docx]

**Supplementary figure and table legends**

**Table S1:** Sequelae for rheumatoid arthritis and the corresponding disability weights in the Global Burden of Disease 2019 Study.

**Table S2**: Prevalence of rheumatoid arthritis in 1990 and 2019 for both sexes and the percentage change in the age-standardised rates (ASRs) per 100,000 in the Middle East and North Africa region (Generated from data available from <http://ghdx.healthdata.org/gbd-results-tool>).

**Table S3:** Incidence of rheumatoid arthritis in 1990 and 2019 for both sexes and the percentage change in the age-standardised rates (ASRs) per 100,000 in the Middle East and North Africa region (Generated from data available from <http://ghdx.healthdata.org/gbd-results-tool>).

**Table S4:** DALYs due to rheumatoid arthritis in 1990 and 2019 for both sexes and the percentage change in the age-standardised rates (ASRs) per 100,000 in the Middle East and North Africa region (Generated from data available from <http://ghdx.healthdata.org/gbd-results-tool>).

**Figure S1:** The percentage change in the age-standardised point prevalence of rheumatoid arthritis in the Middle East and North Africa region from 1990 to 2019, by sex and country. (Generated from data available from <http://ghdx.healthdata.org/gbd-results-tool>).

**Figure S2:** The percentage change in the age-standardised incidence of rheumatoid arthritis in the Middle East and North Africa region from 1990 to 2019, by sex and country. (Generated from data available from <http://ghdx.healthdata.org/gbd-results-tool>).

**Figure S3:** The percentage change in the age-standardised DALYs of rheumatoid arthritis in the Middle East and North Africa region from 1990 to 2019, by sex and country. DALY= disability-adjusted-life-years. (Generated from data available from <http://ghdx.healthdata.org/gbd-results-tool>).
