# Supplementary figures and images for: The burden of rheumatoid arthritis in the Middle East and North Africa region, 1990–2019
Source: Sci Rep. 2022 Nov 11;12:19297. doi: 10.1038/s41598-022-22310-0 (PMC9652423; doi:10.1038/s41598-022-22310-0)

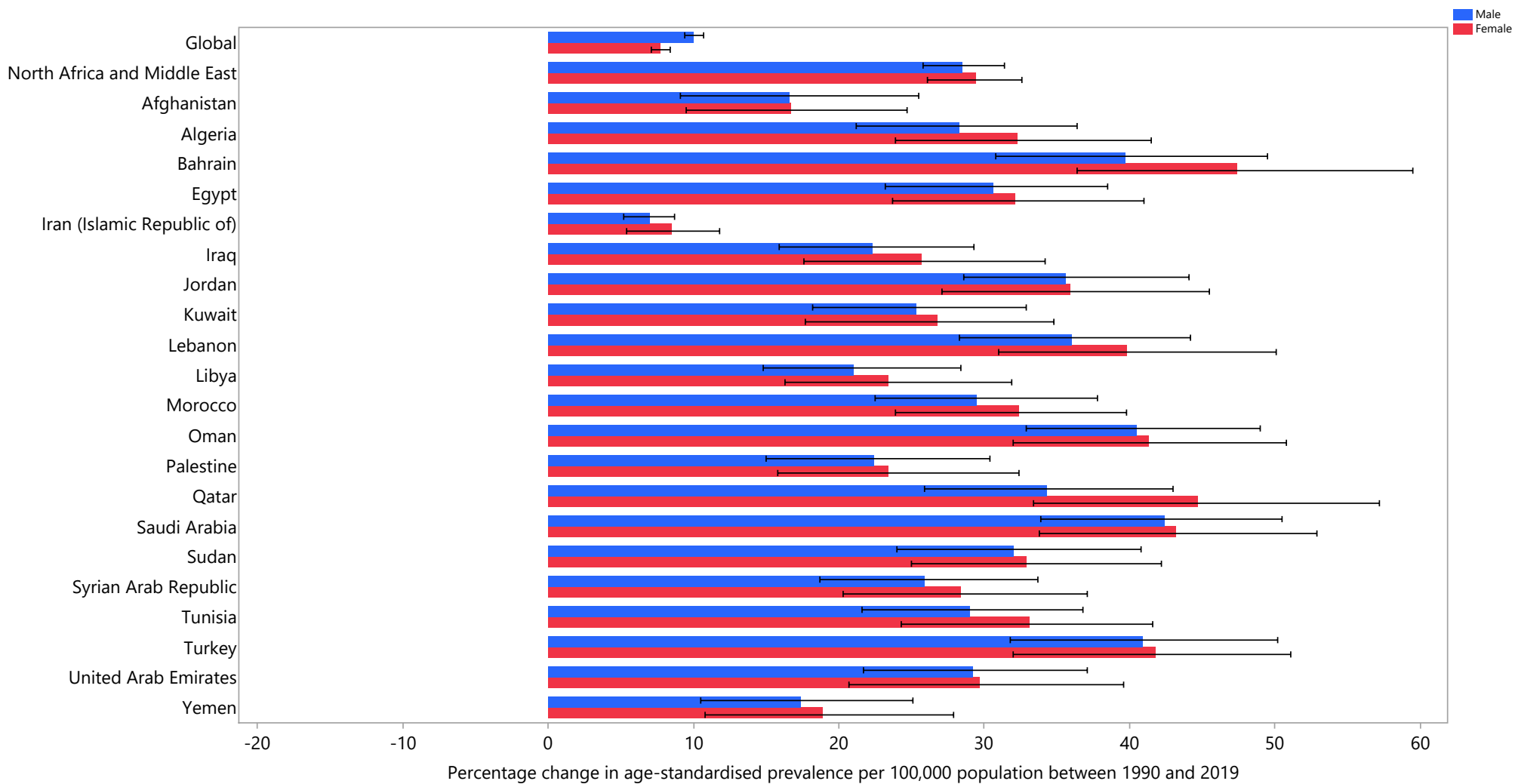

Supplement: Supplementary file 2 — Supplementary Figure S1. [file 41598_2022_22310_MOESM2_ESM.pdf]

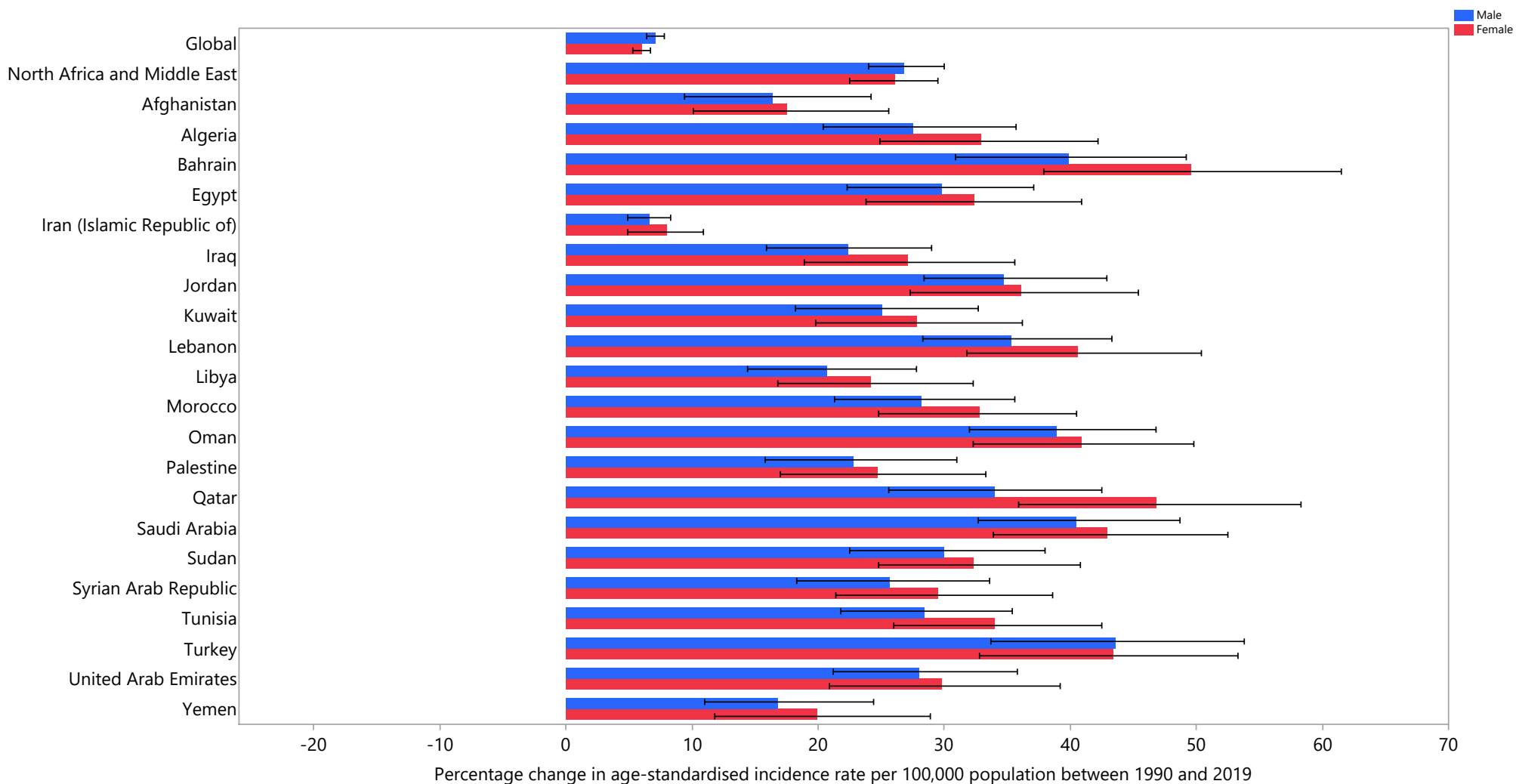

Supplement: Supplementary file 3 — Supplementary Figure S2. [file 41598_2022_22310_MOESM3_ESM.pdf]

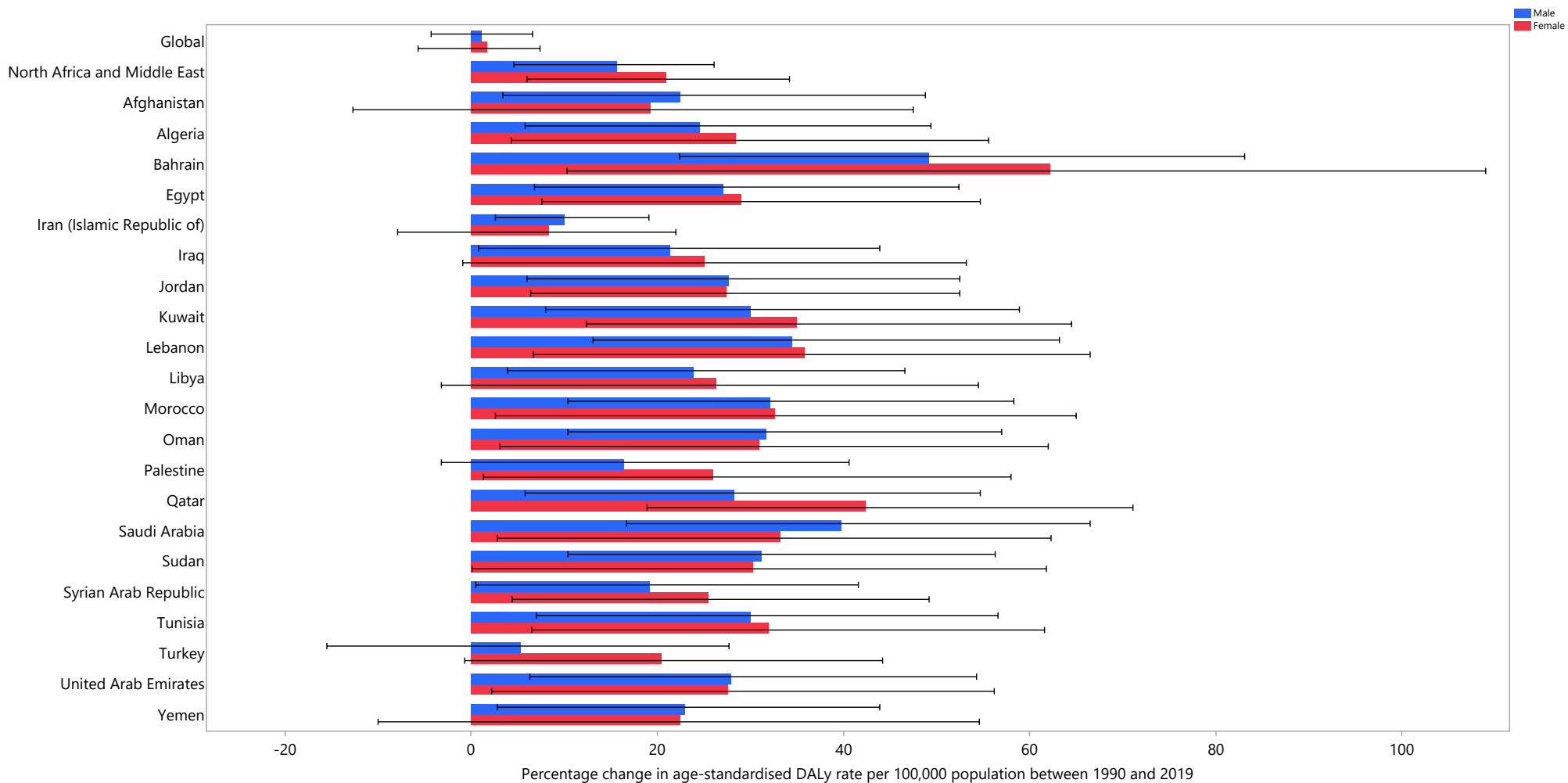

Supplement: Supplementary file 4 — Supplementary Figure S3. [file 41598_2022_22310_MOESM4_ESM.pdf]
