# Supplementary material for: The burden of rheumatoid arthritis in the Middle East and North Africa region, 1990–2019
Source: Sci Rep. 2022 Nov 11;12:19297. doi: 10.1038/s41598-022-22310-0 (PMC9652423; doi:10.1038/s41598-022-22310-0)
Supplement: Supplementary file 5 — Supplementary Table S1. [file 41598_2022_22310_MOESM5_ESM.docx]

| **Table S1: Sequelae for rheumatoid arthritis and the associated disability weights from the Global Burden of Disease 2019 Study** | | |
| --- | --- | --- |
| **Sequela** | **Lay description** | **Disability weight**  **(95% CI)** |
| Mild RA | This person has moderate pain and stiffness in their arms and hands which causes difficulty lifting, carrying, and holding things, and trouble sleeping because of the pain | 0.117  (0.080–0.163) |
| Moderate RA | This person has pain and deformity in most joints, causing difficulty moving around, getting up and down, and using their hands for lifting and carrying. The person often feels fatigue. | 0.317  (0.216–0.440) |
| Severe RA | This person has severe, constant pain, and deformity in most joints, causing difficulty moving around, getting up and down, eating, dressing, lifting, carrying, and using their hands. The person often feels sadness, anxiety, and extreme fatigue. | 0.581  (0.403–0.739) |

Abbreviations: RA: Rheumatoid arthritis; CI: Confidence interval.
