# Supplementary material for: The burden of rheumatoid arthritis in the Middle East and North Africa region, 1990–2019
Source: Sci Rep. 2022 Nov 11;12:19297. doi: 10.1038/s41598-022-22310-0 (PMC9652423; doi:10.1038/s41598-022-22310-0)
Supplement: Supplementary file 6 — Supplementary Table S2. [file 41598_2022_22310_MOESM6_ESM.docx]

| **Table S2: Prevalence of rheumatoid arthritis in 1990 and 2019 for both sexes and the percentage change in the age-standardised rates (ASRs) per 100,000 in the North Africa and the Middle East region**  **(Generated from data available from http://ghdx.healthdata.org/gbd-results-tool)** | | | | | |
| --- | --- | --- | --- | --- | --- |
|  | **1990** | | **2019** | | **Percentage change in ASRs per 100,000** |
|  | **No (95% UI)** | **ASRs per 100,000 (95% UI)** | **No (95% UI)** | **ASRs per 100,000 (95% UI)** |  |
| **Global** | **9019524 (8239786 , 9898679)** | **207.5 (190 , 227)** | **18583481 (16955383 , 20433859)** | **224.2 (204.9 , 246)** | **8.1 (7.5 , 8.6)** |
| **North Africa and Middle East** | **220126 (190013 , 253220)** | **94 (83.1 , 106.7)** | **672827 (588698 , 767998)** | **120.6 (107 , 135.7)** | **28.3 (25.5 , 30.9)** |
| **Afghanistan** | **6093 (5258 , 7135)** | **76.3 (65.9 , 89)** | **19298 (16168 , 23019)** | **87.3 (75.9 , 100.9)** | **14.4 (8.5 , 20.9)** |
| **Algeria** | **13290 (11308 , 15673)** | **80 (69.1 , 92.7)** | **43094 (37004 , 50172)** | **104.2 (90.2 , 120.3)** | **30.3 (24 , 37.3)** |
| **Bahrain** | **398 (333 , 485)** | **113.7 (99.7 , 130.4)** | **2606 (2230 , 3105)** | **154.8 (135.9 , 177.8)** | **36.1 (28.5 , 43.6)** |
| **Egypt** | **30857 (25634 , 37358)** | **75.6 (64 , 89.8)** | **84338 (70531 , 100823)** | **98.1 (83.8 , 115.7)** | **29.8 (23.8 , 36.6)** |
| **Iran (Islamic Republic of)** | **37946 (33742 , 42725)** | **99.8 (89.6 , 110.9)** | **99141 (88570 , 110731)** | **109.1 (97.8 , 120.9)** | **9.3 (7 , 11.8)** |
| **Iraq** | **8884 (7431 , 10715)** | **81.2 (69.5 , 95.3)** | **34227 (28794 , 41102)** | **101.2 (87.1 , 119)** | **24.6 (18.2 , 30.8)** |
| **Jordan** | **1730 (1421 , 2112)** | **76.3 (64.9 , 90.3)** | **10073 (8461 , 12152)** | **102.1 (87 , 120.5)** | **33.8 (27.4 , 40.4)** |
| **Kuwait** | **1174 (948 , 1449)** | **84.4 (70.6 , 100.5)** | **5621 (4652 , 6804)** | **113.3 (96.6 , 133.9)** | **34.2 (27.4 , 40.6)** |
| **Lebanon** | **2118 (1772 , 2522)** | **79.4 (66.8 , 93.5)** | **5965 (5034 , 7036)** | **111.4 (94.3 , 131.1)** | **40.3 (33.8 , 48.1)** |
| **Libya** | **2015 (1653 , 2451)** | **74.5 (62.5 , 88.6)** | **6670 (5544 , 8035)** | **94.1 (79.3 , 111.6)** | **26.3 (20.9 , 32.5)** |
| **Morocco** | **12895 (10754 , 15418)** | **69.3 (59 , 81.6)** | **33404 (28420 , 39495)** | **90.7 (77.3 , 106.8)** | **31 (25 , 36.8)** |
| **Oman** | **862 (708 , 1053)** | **68.8 (58.7 , 81.3)** | **3863 (3166 , 4768)** | **94.6 (80.7 , 111.8)** | **37.4 (31.1 , 43.6)** |
| **Palestine** | **976 (812 , 1163)** | **82.9 (70.9 , 97.2)** | **3561 (2991 , 4281)** | **99 (85.1 , 116.2)** | **19.4 (13.7 , 25.9)** |
| **Qatar** | **286 (231 , 357)** | **78.6 (67.5 , 92.6)** | **3001 (2447 , 3718)** | **103.2 (87.3 , 121.4)** | **31.3 (24.7 , 38.1)** |
| **Saudi Arabia** | **7270 (5890 , 8948)** | **68.9 (57.9 , 82.8)** | **36297 (29909 , 44171)** | **98.9 (83.3 , 117.1)** | **43.5 (37.5 , 50.1)** |
| **Sudan** | **8173 (6736 , 9841)** | **62.9 (53.2 , 73.7)** | **24195 (20326 , 29188)** | **83 (71.6 , 97.1)** | **32 (26 , 38.9)** |
| **Syrian Arab Republic** | **5791 (4772 , 6990)** | **75.3 (63.3 , 88.7)** | **14115 (11938 , 16712)** | **98.7 (83.5 , 116.2)** | **31.1 (25 , 37.8)** |
| **Tunisia** | **4882 (4104 , 5893)** | **76.1 (65 , 89.9)** | **13360 (11354 , 15861)** | **101.2 (86.1 , 120.3)** | **33 (26.5 , 39.8)** |
| **Turkey** | **68619 (61228 , 76578)** | **153.4 (138 , 169.8)** | **203556 (183966 , 223605)** | **217.6 (197 , 238.8)** | **41.8 (33.7 , 49.6)** |
| **United Arab Emirates** | **1022 (814 , 1275)** | **73 (62.1 , 85.7)** | **10061 (8235 , 12561)** | **90.7 (76.8 , 107.6)** | **24.3 (19 , 30.6)** |
| **Yemen** | **4698 (3880 , 5658)** | **62.2 (52.8 , 73.6)** | **15698 (13140 , 18944)** | **73.9 (63.5 , 86.5)** | **18.8 (12.4 , 25.9)** |

Abbreviations: UI: Uncertainty interval; ASR: Age-standardised rate.
