# Supplementary material for: The burden of rheumatoid arthritis in the Middle East and North Africa region, 1990–2019
Source: Sci Rep. 2022 Nov 11;12:19297. doi: 10.1038/s41598-022-22310-0 (PMC9652423; doi:10.1038/s41598-022-22310-0)
Supplement: Supplementary file 7 — Supplementary Table S3. [file 41598_2022_22310_MOESM7_ESM.docx]

| **Table S3: Incidence of rheumatoid arthritis in 1990 and 2019 for both sexes and the percentage change in the age-standardised rates (ASRs) per 100,000 in the North Africa and the Middle East region**  **(Generated from data available from http://ghdx.healthdata.org/gbd-results-tool)** | | | | | |
| --- | --- | --- | --- | --- | --- |
|  | **1990** | | **2019** | | **Percentage change in ASRs per 100,000** |
|  | **No (95% UI)** | **ASRs per 100,000 (95% UI)** | **No (95% UI)** | **ASRs per 100,000 (95% UI)** |  |
| **Global** | **567463 (519417 , 621415)** | **12.2 (11.1 , 13.4)** | **1074391 (975502 , 1179332)** | **13 (11.8 , 14.3)** | **6.5 (5.9 , 7.1)** |
| **North Africa and Middle East** | **13509 (11860 , 15455)** | **4.7 (4.1 , 5.3)** | **36548 (32078 , 41603)** | **5.9 (5.2 , 6.6)** | **25.2 (22.4 , 27.7)** |
| **Afghanistan** | **355 (307 , 416)** | **3.9 (3.4 , 4.5)** | **1338 (1138 , 1586)** | **4.4 (3.8 , 5.1)** | **12 (6.6 , 18)** |
| **Algeria** | **834 (709 , 979)** | **4 (3.4 , 4.6)** | **2294 (1983 , 2670)** | **5.2 (4.6 , 6)** | **31.3 (25.2 , 38.2)** |
| **Bahrain** | **27 (23 , 33)** | **5.7 (5 , 6.5)** | **155 (132 , 182)** | **8.1 (7.1 , 9.2)** | **40.6 (32.4 , 48.9)** |
| **Egypt** | **1847 (1557 , 2205)** | **3.7 (3.2 , 4.4)** | **4763 (4071 , 5605)** | **4.9 (4.2 , 5.7)** | **31.1 (25.1 , 37.2)** |
| **Iran (Islamic Republic of)** | **2314 (2071 , 2597)** | **4.7 (4.2 , 5.2)** | **4684 (4128 , 5303)** | **5 (4.5 , 5.6)** | **7.6 (5.4 , 9.7)** |
| **Iraq** | **568 (483 , 680)** | **4 (3.5 , 4.7)** | **2057 (1767 , 2419)** | **5 (4.3 , 5.8)** | **25.4 (19.7 , 31.7)** |
| **Jordan** | **118 (98 , 144)** | **3.8 (3.2 , 4.4)** | **586 (499 , 693)** | **5 (4.3 , 5.9)** | **34 (27.8 , 40.4)** |
| **Kuwait** | **77 (64 , 95)** | **4.3 (3.6 , 5.1)** | **307 (254 , 373)** | **5.7 (4.9 , 6.7)** | **32.8 (26.2 , 39.1)** |
| **Lebanon** | **115 (98 , 135)** | **3.9 (3.3 , 4.6)** | **289 (246 , 341)** | **5.4 (4.6 , 6.3)** | **38.4 (31.8 , 45.5)** |
| **Libya** | **131 (109 , 157)** | **3.7 (3.1 , 4.4)** | **356 (297 , 426)** | **4.7 (3.9 , 5.5)** | **25.5 (20.5 , 31.3)** |
| **Morocco** | **783 (663 , 925)** | **3.5 (3 , 4)** | **1717 (1475 , 2013)** | **4.5 (3.9 , 5.3)** | **30.7 (25 , 36.7)** |
| **Oman** | **57 (48 , 69)** | **3.4 (2.9 , 4)** | **239 (199 , 295)** | **4.7 (4 , 5.4)** | **37 (31.1 , 42.7)** |
| **Palestine** | **63 (53 , 75)** | **4.1 (3.5 , 4.7)** | **222 (190 , 264)** | **5 (4.3 , 5.7)** | **22.3 (16.3 , 28.8)** |
| **Qatar** | **19 (15 , 23)** | **4 (3.4 , 4.6)** | **185 (151 , 233)** | **5.3 (4.5 , 6.2)** | **33.2 (26.7 , 40.3)** |
| **Saudi Arabia** | **488 (404 , 593)** | **3.5 (3 , 4.1)** | **2111 (1757 , 2575)** | **5 (4.2 , 5.8)** | **42.3 (36.9 , 48.6)** |
| **Sudan** | **523 (443 , 621)** | **3.2 (2.8 , 3.7)** | **1542 (1317 , 1829)** | **4.2 (3.6 , 4.8)** | **31.2 (25.2 , 38)** |
| **Syrian Arab Republic** | **382 (319 , 458)** | **3.8 (3.2 , 4.4)** | **741 (628 , 871)** | **5 (4.2 , 5.9)** | **32.9 (26.6 , 40.2)** |
| **Tunisia** | **287 (243 , 341)** | **3.8 (3.2 , 4.4)** | **633 (541 , 737)** | **5 (4.3 , 5.8)** | **32.8 (26.7 , 39.1)** |
| **Turkey** | **4128 (3709 , 4604)** | **7.9 (7.1 , 8.8)** | **10701 (9656 , 11831)** | **11.4 (10.3 , 12.5)** | **43.1 (34.9 , 51.4)** |
| **United Arab Emirates** | **70 (58 , 87)** | **3.7 (3.2 , 4.3)** | **571 (455 , 718)** | **4.8 (4.1 , 5.6)** | **28.6 (23 , 34.9)** |
| **Yemen** | **314 (265 , 378)** | **3.2 (2.7 , 3.7)** | **1019 (865 , 1212)** | **3.7 (3.2 , 4.4)** | **18.9 (12.9 , 25.5)** |

Abbreviations: UI: Uncertainty interval; ASR: Age-standardised rate.
