# Supplementary material for: The burden of rheumatoid arthritis in the Middle East and North Africa region, 1990–2019
Source: Sci Rep. 2022 Nov 11;12:19297. doi: 10.1038/s41598-022-22310-0 (PMC9652423; doi:10.1038/s41598-022-22310-0)
Supplement: Supplementary file 8 — Supplementary Table S4. [file 41598_2022_22310_MOESM8_ESM.docx]

| **Table S4: DALYs due to rheumatoid arthritis in 1990 and 2019 for both sexes and the percentage change in the age-standardised rates (ASRs) per 100,000 in the North Africa and the Middle East region**  **(Generated from data available from http://ghdx.healthdata.org/gbd-results-tool)** | | | | | |
| --- | --- | --- | --- | --- | --- |
|  | **1990** | | **2019** | | **Percentage change in ASRs per 100,000** |
|  | **No (95% UI)** | **ASRs per 100,000 (95% UI)** | **No (95% UI)** | **ASRs per 100,000 (95% UI)** |  |
| **Global** | **1662622 (1273129 , 2077290)** | **39.1 (30.1 , 48.6)** | **3262589 (2510208 , 4091555)** | **39.6 (30.5 , 49.5)** | **1.1 (-4.3 , 5.2)** |
| **North Africa and Middle East** | **36483 (26371 , 47677)** | **16 (11.6 , 20.9)** | **103572 (74242 , 136655)** | **19 (13.9 , 24.9)** | **18.6 (6.7 , 28.2)** |
| **Afghanistan** | **1051 (737 , 1431)** | **13.2 (9.4 , 17.9)** | **3205 (2238 , 4279)** | **15.8 (11.4 , 20.8)** | **19.7 (-6.6 , 42.1)** |
| **Algeria** | **2026 (1380 , 2760)** | **12.4 (8.5 , 16.8)** | **6391 (4426 , 8496)** | **15.7 (11 , 20.8)** | **26.4 (8 , 46.3)** |
| **Bahrain** | **61 (41 , 83)** | **18.6 (13 , 26.2)** | **421 (302 , 563)** | **27.6 (20.4 , 35.7)** | **48.3 (7.6 , 83)** |
| **Egypt** | **4509 (3042 , 6225)** | **11.2 (7.7 , 15.5)** | **12078 (7916 , 16783)** | **14.2 (9.4 , 19.5)** | **26.2 (8.9 , 45.1)** |
| **Iran (Islamic Republic of)** | **5863 (4191 , 7734)** | **15.8 (11.4 , 20.6)** | **15366 (10877 , 20209)** | **17.3 (12.4 , 22.7)** | **9.8 (-3 , 20.5)** |
| **Iraq** | **1400 (957 , 1903)** | **13 (9 , 17.5)** | **5248 (3610 , 7185)** | **16.1 (11.3 , 21.8)** | **23.9 (3.5 , 45.4)** |
| **Jordan** | **267 (182 , 367)** | **12.3 (8.6 , 16.5)** | **1487 (997 , 2044)** | **15.5 (10.5 , 20.8)** | **25.6 (9.5 , 44.2)** |
| **Kuwait** | **164 (106 , 228)** | **11.7 (7.8 , 16.2)** | **797 (524 , 1109)** | **16.5 (11.2 , 22.7)** | **40.8 (23.1 , 60.9)** |
| **Lebanon** | **321 (217 , 441)** | **12.2 (8.3 , 16.5)** | **894 (628 , 1211)** | **16.7 (11.8 , 22.5)** | **37.4 (14.8 , 61.7)** |
| **Libya** | **302 (204 , 413)** | **11.4 (7.8 , 15.4)** | **1012 (700 , 1373)** | **14.7 (10.3 , 19.6)** | **29 (6.7 , 48.4)** |
| **Morocco** | **1998 (1357 , 2766)** | **11 (7.5 , 15.2)** | **5255 (3658 , 7094)** | **14.6 (10.2 , 19.5)** | **32 (9.4 , 55.8)** |
| **Oman** | **132 (89 , 183)** | **11.2 (7.8 , 15)** | **559 (376 , 762)** | **14.3 (10 , 18.7)** | **27.3 (6.9 , 47.9)** |
| **Palestine** | **159 (112 , 216)** | **14 (10.1 , 18.7)** | **566 (396 , 755)** | **16.8 (11.9 , 22.1)** | **20.4 (1.4 , 42.9)** |
| **Qatar** | **41 (27 , 58)** | **12.1 (8.3 , 16.3)** | **421 (280 , 592)** | **15.1 (10.6 , 20.6)** | **25.2 (9.6 , 44.1)** |
| **Saudi Arabia** | **1072 (718 , 1497)** | **10.5 (7.1 , 14.5)** | **5140 (3389 , 7175)** | **14.2 (9.7 , 19.5)** | **35.9 (14 , 55.4)** |
| **Sudan** | **1302 (895 , 1736)** | **10.3 (7.2 , 13.7)** | **3752 (2637 , 5138)** | **13.4 (9.6 , 17.9)** | **29.7 (7.8 , 52.1)** |
| **Syrian Arab Republic** | **878 (606 , 1203)** | **11.6 (8.1 , 15.7)** | **2073 (1416 , 2802)** | **14.7 (10.1 , 19.7)** | **26.4 (10 , 45.8)** |
| **Tunisia** | **726 (493 , 1005)** | **11.4 (7.8 , 15.8)** | **1993 (1378 , 2743)** | **15.2 (10.4 , 20.8)** | **32.7 (13.8 , 54.5)** |
| **Turkey** | **13295 (9917 , 17156)** | **30.5 (22.9 , 39.4)** | **32813 (24231 , 42624)** | **35.4 (26.2 , 45.9)** | **16.2 (-1.9 , 34.6)** |
| **United Arab Emirates** | **150 (99 , 212)** | **11.5 (8.1 , 15.6)** | **1484 (973 , 2034)** | **14 (9.6 , 18.5)** | **21.5 (5.2 , 39.3)** |
| **Yemen** | **740 (499 , 1013)** | **10.2 (7.1 , 13.8)** | **2511 (1735 , 3350)** | **12.5 (8.9 , 16.4)** | **22.5 (-2.8 , 45.3)** |

Abbreviations: DALY: Disability-adjusted life-years; UI: Uncertainty interval; ASR: Age-standardised rate.
